# Prevalence and predictors of prior antibacterial use among patients presenting to hospitals in Northern Uganda

**DOI:** 10.1186/s40360-015-0027-8

**Published:** 2015-09-25

**Authors:** Moses Ocan, Yukari C. Manabe, Hannington Baluku, Esther Atukwase, Jasper Ogwal-Okeng, Celestino Obua

**Affiliations:** Department of Pharmacology & Therapeutics, Makerere University, P.O. Box 7072, Kampala, Uganda; Department of Clinical Microbiology, Makerere University, P.O. Box 7072, Kampala, Uganda; Mild may Uganda, P. O. Box 24985, Kampala, Uganda; Faculty of medicine, Gulu University, P.O. Box 166, Gulu, Uganda; Division of Infectious Diseases Department of Medicine, Johns Hopkins School of Medicine, Baltimore, USA

**Keywords:** Antibacterial, Urine antibacterial bioassay, Self-report, Self-medication, Northern Uganda

## Abstract

**Background:**

Human antibacterial exposure occur in different ways including consumption of animal and agricultural products as well as use of prescribed and non-prescribed agents. We estimated the prevalence and explored the predictors of antibacterial use among patients presenting to hospitals in northern Uganda.

**Methods:**

Four hundred fifty (450) patients were randomly selected and antibacterial use prior to hospital visit measured using a questionnaire and urine antibacterial activity assay. Urine antibacterial bioassays were performed using American type culture collections of *Escherichia coli*, *Bacillus subtilis* and *Streptococcus pyogenes*. Data were analysed using STATA 12.0 at 95 % confidence level.

**Results:**

Of 450 patients interviewed, 62.2 % had used antibacterial agents. Urine antibacterial activity was detected in 30.4 % of the samples tested. Of the 85 patients who reported not taking any antibacterial at home, 16 (18.8 %) had urine with antibacterial activity. Most test bacteria, *E. coli* (74.5 %), *B. subtilis* (72.6 %) and *S. pyogens* (86.7 %) were sensitive to urine of patients who reported using antibacterial drugs before hospital visit. From the interview, metronidazole 15.6 % (70/450), amoxicillin 12 % (54/450), and ciprofloxacin 10.4 % (47/450) were the most used antibacterial agents. Patient age (OR, 2.45: 95 % CI: 1.02–5.91: *P* = 0.024), time-lag between last drug intake and hospital visit (OR: 3.18: 95 % CI: 1.44–7.0: *P* < 0.0001), and time-lag between illness onset and hospital visit (OR: 1.89: 95 % CI: 0.38–5.1: *P* = 0.027) predicted the use of antibacterial agents before hospital visit.

**Discussion:**

Community antibacterial use continues to take place in an unregulated manner. In addition, physiciansrarely seek to ascertain prior use of antibacterial agents among patients presenting to hospitals. This couldhave a bearing on patient treatment outcomes.

**Conclusion:**

Knowledge of prior antibacterial use among patients presenting to hospitals is useful to physicians in ensuring antibacterial stewardship.

## Background

Antibacterial use both in hospital settings and different communities of the world is increasing exponentially. A study [1] found that there was a 36 % total increase in consumption of antibacterial agents globally from 2000 to 2010. The increased global burden of infectious diseases may explain the rise in use of antibacterial drugs globally [2, 3]. In low income countries, easy over-the-counter access due to inadequate enforcement or lack of laws restricting non-prescription sale of antibacterial agents further influence the increased volume of antibacterials consumed [1–4]. This rise in global use of antibacterial agents has been found to be associated with corresponding increase in development and spread of resistance [5, 6].

The use of antibacterial agents prior to hospital visit is common especially in developing countries and has the potential of influencing patient treatment outcomes [6, 7]. Assessment of body fluids like plasma using chromatographic methods such as high performance liquid chromatography (HPLC) can help in establishing prior drug use among patients. However the high associated cost in addition to the technical expertise required limits its use as a point of care technique for establishing prior antibacterial use especially in resource constrained countries like Uganda. In most developing countries, health workers rely on self-report in assessing antibacterial use among patients prior to hospital visit, information critical for appropriate therapeutic choices. The high illiteracy levels in addition to recall bias reduces the effectiveness of self-report [8–10]. Previous studies have reported cheap and reliable method of measuring prior antibacterial use among patients who visit hospitals in developing countries using urine antibacterial activity bioassay [11, 12].

The use of non-prescription antibacterial agents before seeking professional medical treatment is widespread in most communities Worldwide [4]. In this study, we assessed the prevalence and predictors of prior antibacterial use among out-patients in northern Uganda using questionnaire technique and urine antibacterial bioassay.

## Methods

### Ethics statement

The study protocol was reviewed and approved by the School of Medicine, Makerere University research and ethics review committee (REC REF 2012–072), and Uganda National Council for Science and Technology (HS 126). Permission was obtain from the administration of the two hospitals. All selected patients provided written informed consent.

### Study design, site and population

This was a cross sectional study among adult (≥18 years) patients presenting to general out-patient departments of Lira and Gulu Regional Referral Hospitals (RRHs) in northern Uganda. Lira RRH is a 415 bed public hospital serving a population of over 2.5 million people and is located in central northern Uganda about 375 km from the capital city Kampala. The hospital receives approximately 131,296 patients annually in the general out-patient department. Gulu RRH is a 397 bed public hospital serving a population of over 1.5 million people and is located about 364 km from the capital city Kampala. The hospital receives approximately 77,128 patients annually in the general out-patient department.

### Sampling criteria

For each day of data collection, the first patient to be recruited into the study was randomly chosen from among the patients waiting to be seen in the general outpatient departments using systematic random sampling. In Lira and Gulu RRHs, intervals of 14 and 23 were used respectively to randomly select subsequent patients to approach for recruitment in the study.

### Questionnaire and data collection

Data collection for the study was carried out in 3 months from August-to-October 2013 in northern Uganda. Data on use of antibacterial agents at home prior to hospital visit was collected using a structured interviewer administered questionnaire. Information from previous studies was used in developing the data collection tool. The tool was pre-tested on 20 patients presenting to out-patient department of mulago national referral hospital in Kampala city. The questionnaire was administered by four trained pharmacy technicians of Lira and Gulu regional referral hospitals. The questions which were asked included; i) What is your age, occupation, sex, and level of education?, ii) How long did you take since you first felt the symptoms of the current illness before you came to the hospital today?, iii) Before coming to the hospital today, did you take any medicine at home for the current illness?, iv) If yes, why did you first treat yourself at home before coming to the hospital?, v) What was the name of the medicine(s) which you were taking before coming to the hospital?, vi) What was the color and formulation (tablet, capsule or others) of the medicine (s) which you took at home before coming to the hospital?, vii) If more than one, were you taking them concurrently?, viii) What was the name of the disease symptom that the antibacterial drug was used to manage?, ix) When did you last take this antibacterial agent (s) before coming to the hospital?, x) Who initiated or recommended the use of this antibacterial agent (s) that you took at home prior to hospital visit?, xi) What was the source of the antibacterial agent (s) that you took at home before coming to the hospital today?, xii) Did you take more than one type of antibacterial agent to treat yourself at home before coming to the hospital today?.

The interviews lasted between 15–30 min per patient. The patients were then requested to collect spontaneously voided urine in wide mouth sterile containers. The urine antibacterial activity bioassays were performed immediately upon receipt of the sample.

### Urine antibacterial bioassay

Urine antibacterial bioassay was performed using a modified method [11], *Bacillus subtilis* was used instead of *B. stearothermophilus* due to the ease of incubation of *B. subtilis* as opposed to *B. stearothermophilus*. Two culture media; Mueller Hington II (MHT 20500) agar (*Escherichia coli* and *Bacillus subtilis*) and 5 % sheep blood agar (*Streptococcus pyogenes)* were used to determine the inhibitory activity of patients’ urine against standard bacterial strains obtained from the American Type Culture Collection (ATCC; Rockville). Standard discs (6 mm diameter) were cut from Whatman filter paper No.1 and autoclaved together with the cotton tipped swabs for 15 minutes at 121 °C. Lyophilized test organisms, *Bacillus subtilis*, *E. coli* and *S. pyogenes* were cultured. Bacterial colonies from each of the cultured standard organisms were suspended in 0.85 % sodium chloride solution and the turbidity adjusted to 0.5 McFarland units. A sterile cotton tipped swab was dipped in the bacterial suspension and evenly streaked onto the culture media for the respective reference organisms. Each Petri dish was marked with numbers 1, 2, 3, 4 and 5 (all along the outer edge of the plate) and 6 at the centre. The plain sterile filter paper discs were completely immersed in urine using sterile forceps. Excess urine was removed and the discs firmly pressed onto the agar surface corresponding to the marked positions. The five positions marked on the outer edges of each petri dish corresponded to urine for different patients. Each sample was plated in duplicate on separate petri dishes. A blank filter paper disc was pressed at position 6 in the centre of both petri dishes as a negative control. The plates were then incubated at 35–37 °C for 24 h after which the zones of inhibition were measured. The zone diameter was read as zero if bacterial growth extended up to the disk. If one or both duplicate filter paper discs for each reference organism had a visible zone of inhibition the patient was recorded as having urinary antibacterial activity. Urine samples with visible signs of faecal contamination were excluded from the bioassay. The diameters of the zone of inhibition were measured using a tape measure and expressed as the mean of duplicate readings [13]. Urine antibacterial activity was defined as presence of a zone of inhibition in any one of the duplicate urine culture plates.

A control media plate was incubated at 37 °C overnight to check for sterility before the plates were used. Fresh suspensions of the ATCC indicator bacteria (ATCC 6051, ATCC 25922, and ATCC 19615) were made and used each time to avoid contamination. At the beginning of the study, the indicator organisms were subjected to the commonly used antibacterial agents; ampicillin, ciprofloxacin, gentamicin, augmentin, cotrimoxazole, chloramphenicol, tetracycline, penicillin, erythromycin and ceftriaxone. Kirby-Bauer disc diffusion method on Mueller Hington agar following CLSI guidelines was used [14].

### Data management

Double data entry of questionnaire information was done using Epi Info 3.5.2 (Epi Info, National Centre for Public Health Informatics, Centres for Diseases Control, USA). Laboratory data on the diameters of the zones of inhibition was also entered in duplicate in Excel spread sheet, 2007. The entries were then merged together in STATA 12.0. Any discrepancies in the entries were resolved by referring to the source documents.

### Statistical analysis

Proportions, means and standard deviations were used to describe the study characteristics. The dependent variable was urine antibacterial activity. Factors associated with urine antibacterial activity among the patients were estimated using chi square statistics for categorical variables and *t*-test for continuous variables.

Logistic regression was used to determine the predictors of urine antibacterial activity among the patients presenting to general out-patient departments of Lira and Gulu RRHs in northern Uganda. The independent variables considered included, socio-demographic factors, self-reported antibacterial use, time of last antibacterial intake prior to hospital visit, time-lag between the onset of disease symptoms and hospital visit, the kind of antibacterial drug used before hospital visit and how it was taken. The proportions of responses were estimated using STATA 12.0. In uni-variable models, each independent variable was regressed with the outcome variable to determine the crude associations at 95 % level of significance.

All factors which were significant (*P* < 0.05) were considered in the multivariable model to determine joint predictors of urine antibacterial activity. The model was built using forward fitting algorithm and goodness-of-fit determined using Hosmer-Lemeshow test. Wald test was performed to determine the joint significance of the explanatory variables in the final multivariable model.

Patients who reported having used only metronidazole were excluded from the estimation of the correlation between questionnaire technique and urine antibacterial activity. This is because their urine samples did not show any antibacterial activity against the ATCC bacterial strains used in the bioassay [15].

## Results

### Socio-demographic characteristics of the respondents

In this study, 450 participants were interviewed and each provided a urine sample for the antibacterial activity bioassay. The majority of respondents (342/450, 76 %) were females. The mean age of the participants was 33.3 ± 13.6 years. One hundred thirty six of the respondents, 30.2 % (136/450) were engaged in unskilled labour and 48.4 % (218/450) lacked formal education (Table 1).

**Table 1:** Demographic characteristics of the respondents and urine antibiotic activity

Factor	Description	Number of participants, (*N* = 450; %)	Number of urine samples with antibiotic activity (*N* = 137; %)	*P*-value
Sex	Female	342 (76 %)	106 (77.4 %)	0.652
	Male	108 (24 %)	31 (22.6 %)	
Age (years)	18–27	199 (44.2 %)	43 (31.4 %)	0.008
	28–37	102 (22.7 %)	38 (27.7 %)	
	38–47	81 (18 %)	32 (23.4 %)	
	48–57	34 (7.6 %)	(9.5 %)	
	≥58	34 (7.6 %)	11 (8.0 %)	
Occupation	Unskilled labour	136 (30.2 %)	48 (35.0 %)	0.371
	Professional	87 (19.3 %)	22 (16.1 %)	
	Business owner	48 (10.7 %)	17 (12.4 %)	
	Others	179 (39.8 %)	50 (36.5 %)	
Education level	None	218 (48.4 %)	77 (56.2 %)	0.129
	Primary level	131 (29.1 %)	37 (27 %)	
	Secondary level	52 (11.6 %)	16 (11.7 %)	
	Tertiary level	49 (10.9 %)	7 (5.1 %)	

### Antibacterial agents used by participants prior to coming to the hospital

The commonly used antibacterial agents included metronidazole 15.6 % (70/450), amoxicillin 12 % (54/450), ciprofloxacin 10.4 % (47/450), doxycycline 6 % (27/450) and cotrimoxazole 4.9 % (22/450). Public health facilities 51.8 % (233/450), drug shops/pharmacies 31.3 % (141/450) and clinics 16.7 % (75/450 were the major sources of antibacterial agents used. The advice for using antibacterial agents prior to hospital visit was commonly obtained from health professionals 71.8 % (323/450), drug sellers 18.9 % (85/450), self 8.4 % (38/450) and household member 4.2 % (19/450).

### Prevalence and factors associated with use of antibacterial agents prior to hospital visit

Of the 450 respondents, 62.2 % (280) reported using antibacterial agents from home before visiting the hospital. The most reported disease symptoms which led to home treatment were mild illness with (112/450: 24.9 %) or without (238/450: 52.9 %) fever. While, successful experience in treatment of previous illness was the most common reason (130/450: 28.9 %) for taking antibacterial drugs at home prior to hospital visit.

### Urine antibacterial bioassay against *E. coli, B. subtilis* and *S. pyogenes*

Antibacterial activity was demonstrated on at least one of the three standard test bacterial strains in 30.4 % (137/450) of the urine samples. The growth of *Bacillus subtilis* was inhibited by 25.1 % (113/450) of the urine samples with the mean zone of inhibition being 3.6 ± 6.9 mm. Most of the urine samples with antibacterial activity against *B. subtilis,* 85.8 % (97/113) had a smaller zone of inhibition (1 mm), and 12.4 % (14/113) had a medium zone of inhibition (21 mm) while 1.8 % (2/113) had a larger zone of inhibition (≥31 mm). For *E. coli,* growth was inhibited by 20.9 % (94/450) of the urine samples with mean inhibition zone diameter of 2.8 ± 6.4 mm. Of the urine samples with antibacterial activity against *E. coli,* 80.9 % (76/94) had a smaller zone of inhibition, 18.1 % (17/94) had a medium zone of inhibition while 1.1 % (1/94) had a larger zone of inhibition. For *S. pyogenes* growth was inhibited by 16.7 % (75/450) of the urine samples with mean inhibition zone diameter of 3.2 ± 8 mm. Of the urine samples with antibacterial activity against *S. pyogenes,* 56 % (42/75) had a smaller zone of inhibition, 34.7 % (26/75) had a medium zone of inhibition while 9.3 % (7/75) had a larger zone of inhibition.

**Fig. 1:**
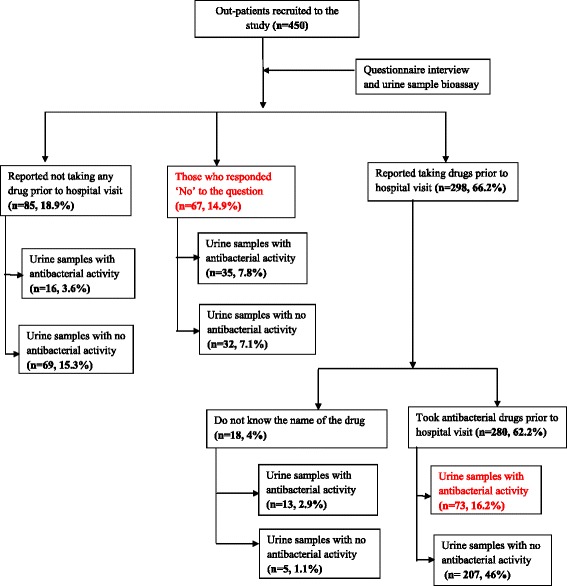
Study flow chart

The patients who reported use of antibacterial drugs prior to hospital visit had more urine samples with higher zones of inhibition than those who did not report use of antibacterial drugs before visiting the hospital. Among the patients who reported taking antibacterial drugs prior to hospital visit and who had a positive urine antibacterial assay (*n* = 137), 62 % (85) reported using more than one antibacterial agent at home prior to visiting the hospital. Thirty six (8 %) urine samples had antibacterial activity on only one organism, 57 (12.7 %) on two organisms while 44 (9.8 %) were positive on all three strains of the test organisms. Of the 85 urine samples from patients who reported not to have taken antibacterial agents prior to hospital visit, 18.8 % (16/85) had positive antibacterial activity (Fig. 1).

### Factors associated with urine antibacterial activity

In the multivariable model, the age of study participants was significantly associated with urine antibacterial activity with those who were 48–57 years of age had two times more positive urine antibacterial activity compared to 18–27 year old participants (OR, 2.45: 95 % CI: 1.02–5.91: *P* = 0.024). Patients who reported taking medicines at home on the same day of hospital visit had three times more urine antibacterial activity (OR: 3.18, 95 % CI: 1.4–7.0, *P* < 0.0001) than those who did not (Table 2). The model fitted with Hosmer-Lemeshow (H-L) probability of 0.92 (*P* = 0.92) on ten (10) groups.

**Table 2:** Factors associated with urine antibacterial activity

Variable	Description	Proportion with urine activity (*n* = 137)	Crude OR (95%CI)	Adjusted OR (95%CI)	*P*-value
Patient age (years)	18–27	43 (31.4 %)	1.0	1.0	0.024
	28–37	38 (27.7 %)	2.15 (1.27–3.64)	2.20 (1.24–3.90)	
	38–47	32 (23.4 %)	2.37 (1.35–4.14)	2.23 (1.20–4.13)	
	48–57	13 (9.5 %)	2.25 (1.04–4.45)	2.45 (1.02–5.91)	
	58+	11 (8.1 %)	1.74 (0.78–3.84)	2.02 (0.81–5.01)	
Duration of time of last drug intake prior to hospital visit	Did not take any antibacterial	16 (11.7 %)	1.0	1.0	<0.0001
	Today	51 (37.2 %)	4.07 (2.09–7.92)	3.18 (1.44–7.00)	
	2–4 days ago	45 (32.8 %)	2.81 (1.45–5.45)	1.81 (0.82–4.03)	
	5–7 days ago	1 (0.7 %)	0.25 (0.03–2.05)	0.16 (0.02–1.37)	
	8–30 days ago	18 (13.1 %)	1.92 (0.86–4.27)	1.40 (0.55–3.55)	
	Do not remember	16 (11.7 %)	0.51 (0.20–1.26)	0.41 (0.16–1.08)	
Duration of time between first illness onset and hospital visit	Today	3 (2.2 %)	1.0	1.0	0.027
	2 days ago	5 (3.6 %)	0.21 (0.04–1.12)	0.1 (0.08–0.57)	
	A week ago	36 (26.3 %)	2.1 (0.31–4.2)	1.03 (0.25–4.2)	
	More than a week	93 (67.9 %)	2.4 (0.47–6.0)	1.89 (0.38–5.1)	
Used more than one type of drug at home prior to hospital visit	No	12 (9.8 %)	1.0	1.0	0.05
	Yes	61 (44.5 %)	2.46 (1.63–3.71)	1.6 (0.9–2.7)	

*OR* Odds Ratio, *CI* Confidence Interval

In all the urine culture plates there was no observable inhibition of bacterial growth by the blank filter paper discs which were pressed at the centre of each plate as negative controls during the antibacterial activity bioassay. In addition, the ATCC bacterial strains used in the urine antibacterial activity bioassays were all sensitive to the commonly used antibacterial agents.

## Discussion

The use of antibacterial drugs prior to hospital visit is a potential threat to patient care and is a common practice in many resource limited communities globally. In this study, a third of the urine samples tested had positive antibacterial activity indicating prior antibacterial exposure among out-patients presenting to the hospitals in northern Uganda. This finding is similar to that of a previous study done in Nepal [16]. The high prevalence of infectious diseases especially in developing countries, coupled with easy access over-the-counter influences the volume of antibacterial agents consumed [2, 17]. A previous study [18] found a high prevalence of non-prescription use of antibacterial agents in communities of northern Uganda. This coupled with the high prescription of antibacterials in hospitals especially in low income countries in addition to potential exposure through consumption of animal and agricultural products creates drug pressure in communities which potentially increase the risk of resistance development [5, 17, 19]. Although antibacterial resistance can develop irrespective of whether the drug is used appropriately or not, the risk is likely to be higher when used inappropriately, a practice common in self-medication [20, 21]. Establishing trends in antibacterial consumption is key in understanding the epidemiology of resistance. This can help predict the threat of resistance in patient care, establish initiatives to preserve efficacy of antibacterial agents and provide baseline data for the assessment of efforts for future reduction in community consumption of antibacterial agents [1, 22, 23].

The high proportion of female respondents found in this study could be attributed to their generally better healthcare seeking behaviour [24]. Earlier studies have reported past experiences as a main determinant of antibacterial self-medication especially in resource limited countries [25, 26]. In this study, the likelihood of taking antibacterial agent prior to hospital visit increased with increase in age beyond 18 years. This could be due to the accumulated experiences in the treatment of infectious diseases which are highly prevalent in this settings [23]. The longer the time-lag between reported onset of illness and hospital visit, the more likely for patients to have positive urine antibacterial activity indicating prior exposure. Most patients spent more than a week taking antibacterial drugs at home before visiting the hospital. A third of the deaths (33 %) in a South African study associated to malaria were due to delays of three or more days before seeking professional medical treatment [21].

A previous study [27] reported that patients were generally unwilling to disclose information on their non-prescription use of antibacterial agents. In addition, physicians rarely explore to establish existence of prior antibacterial use among patients presenting to hospitals for healthcare. This was shown by our findings in which some patients who reported not to have taken any antibacterial prior to hospital visit were found to have urine with antibacterial activity. Patients are thus likely to be prescribed antibacterial agents which they have already tried at home before coming to the hospital. This is likely especially due to the limited therapeutic choices in this settings and could increase the risk of treatment failure which potentially affects patient trust and confidence in the physicians and the healthcare system. Therefore assessment of antibacterial use prior to hospital visit among patients is a useful practice in ensuring antibacterial stewardship. This can be achieved using both qualitative and quantitative methods. The use of urine antibacterial bioassay is a valuable and affordable tool for assessment of prior antibacterial use among patients presenting to hospital before initiating any treatment especially in resource limited communities [11].

The study found that questionnaire technique estimated a higher prevalence of antibacterial use prior to hospital visit among out-patients compared to urine antibacterial assay, contrary to findings of previous studies [11]. Most patients reported to have used metronidazole, amoxicillin, ciprofloxacin, doxycycline and cotrimoxazole prior to hospital visit. This was consistent with the bioassay findings in which more urine samples from patients who reported to have taken antibacterial drugs had antibacterial activity with larger zones of inhibition. The absence of antibacterial activity of the urine samples of some patients who reported to have taken antibacterial drugs prior to hospital visit could be attributed to the uncertainty between actual dose, dosing time and when sample collection was performed. In addition, the lack of awareness by patients regarding the exact kind of medicine which was taken may have contributed to the urine antibacterial bioassay findings in this study. Furthermore, the quality of antibacterial drugs may also affect the urine antibacterial activity as high prevalence of substandard antimicrobial agents especially in developing countries has been previously reported [28]. The high level of illiteracy in northern Uganda, a region which suffered more than two decades of armed conflict potentially affects patients recall in addition to inadequate labelling of packaging materials from drug outlets [8, 29, 30]. The findings of our study are useful to physicians, the community and policy makers as it provides evidence of prior antibacterial use among patients seeking treatment in hospitals and can be used in informing antibacterial stewardship strategies among health professionals and the communities in northern Uganda.

The study had some limitations high level of illiteracy in the region might have affected patient recall. Urine antibacterial activity depends on rate, extent of urine excretion, the timing of urine collection and is dependent on the sensitivity of ATCC bacterial strains used against commonly used antibacterial agents. Measurement of actual antibacterial drug concentrations in blood or urine using HPLC would be a preferable method of establishing prior antibacterial use however associated high cost and technical requirements would limit its application at point of care for routine assessment of prior antibacterial use especially in resource limited settings of developing countries.

## Conclusion

A third of the patients presenting to the general out-patient departments of hospitals in northern Uganda have taken antibacterial agents prior to hospital visit. Knowledge of prior antibacterial use among patients presenting to hospitals is useful to physicians in ensuring antibacterial stewardship.

## Abbreviation

CLSI: Clinical Laboratory Standards Institute
ATCC: American type culture collection
HPLC: High performance liquid Chromatography
RRH: Regional Referral Hospital
REC REF: Review committee reference
OR: Odds ratio
CI: Confidence interval
